# A Sensorimotor Signature of the Transition to Conscious Social Perception: Co-regulation of Active and Passive Touch

**DOI:** 10.3389/fpsyg.2017.01778

**Published:** 2017-10-13

**Authors:** Hiroki Kojima, Tom Froese, Mizuki Oka, Hiroyuki Iizuka, Takashi Ikegami

**Affiliations:** ^1^Graduate School of Arts and Sciences, The University of Tokyo, Tokyo, Japan; ^2^Institute for Applied Mathematics and Systems Research (IIMAS), National Autonomous University of Mexico (UNAM), Mexico City, Mexico; ^3^Center for Complexity Sciences (C3), National Autonomous University of Mexico (UNAM), Mexico City, Mexico; ^4^Graduate School of Systems and Information Engineering, University of Tsukuba, Ibaraki, Japan; ^5^Graduate School of Information Science and Technology, Hokkaido University, Sapporo, Japan

**Keywords:** embodied cognition, social interaction, intersubjectivity, agency detection, direct perception

## Abstract

It is not yet well understood how we become conscious of the presence of other people as being other subjects in their own right. Developmental and phenomenological approaches are converging on a relational hypothesis: my perception of a “you” is primarily constituted by another subject’s attention being directed toward “me.” This is particularly the case when my body is being physically explored in an intentional manner. We set out to characterize the sensorimotor signature of the transition to being aware of the other by re-analyzing time series of embodied interactions between pairs of adults (recorded during a “perceptual crossing” experiment). Measures of turn-taking and movement synchrony were used to quantify social coordination, and transfer entropy was used to quantify direction of influence. We found that the transition leading to one’s conscious perception of the other’s presence was indeed characterized by a significant increase in one’s passive reception of the other’s tactile stimulations. Unexpectedly, one’s clear experience of such passive touch was consistently followed by a switch to active touching of the other, while the other correspondingly became more passive, which suggests that this intersubjective experience was reciprocally co-regulated by both participants.

## Introduction

There is growing acceptance that humans develop social awareness much earlier than had long been assumed, including suggestions of a capacity for false belief understanding even in the case of preverbal infants (Baillargeon et al., 2010). Moreover, there is compelling evidence for the ability to recognize and respond to another’s presence appropriately even in the first months after birth (Reddy, 2008). In order to account for these findings it is necessary to expand the explanatory framework that has traditionally been employed by developmental psychology and social cognition research (Hutto et al., 2011). The root developmental form of social understanding is starting to be conceived as more interactive and perceptual than just detached and cognitive. For example, before infants can begin to theorize about another person’s minds from a third-person perspective, or to imagine what it is like to be them from a first-person perspective, they arguably can already experience another’s presence from the second-person perspective, that is, in the context of mutual engagement (Reddy and Morris, 2004). On this expanded view, the classic mechanisms of social cognition build on and are preceded by embodied forms of social understanding, such as those realized by neural resonance in motor areas (Gallese, 2007) and by the dynamics and experience of social coordination itself (Froese and Gallagher, 2012). However, although the “higher-level” cognitive capacities develop on the basis of the “lower-level” embodied capacities, they do not replace them. Development is characterized by a diversification of social capacities, and we learn to deploy a combination of both embodied and cognitive skills in a context-sensitive manner (Fiebich et al., 2016).

This reappraisal of the development of social awareness is consistent with an ongoing reevaluation of social understanding in adults. It is already widely accepted that our capacities for theorizing about and simulating other minds are not mutually exclusive but complementary (Frith and Frith, 2012), for example in terms of dual processing (Bohl and van den Bos, 2012). And, as has been argued by phenomenological, embodied, and enactive approaches, it makes sense that the mechanisms of reflective social cognition are in turn complemented by interaction dynamics and perceptual experience (Wiltshire et al., 2015). In contrast, it had long been assumed that social understanding must be mainly cognitive because other minds are fundamentally unobservable (Premack and Woodruff, 1978), a claim which follows from the traditional theory that we only perceive physical properties of the world. However, this position is problematic because it underestimates the scope of perceptual experience and is therefore forced to overburden the role of intellectual activity (Gallagher, 2008). When we attend to how we relate to other persons in our everyday life, we realize that the ability to simply perceive others as subjects guided by their own minds remains our primary means of social understanding and that uncertainties can often be resolved in interaction (Gallagher, 2012).

This phenomenological insight is particularly evident when adopting a second-person perspective, that is, when we are engaged in mutually responsive interaction in which another person appears as an immediate “you” rather than as a detached he or she. As long as there is nothing strange about the situation, it is possible to perceive a large extent of the other’s psychological states in the ways in which they act in the world and especially in how they interact with us (Ratcliffe, 2007). In acknowledgment of ecological theories of perception, this capacity to perceive the other’s mind without the need for explicit theorizing or imagining is often referred to as the “direct perception” of other minds (Froese and Leavens, 2014). As the direct social perception thesis has become more widely accepted in cognitive science, the debate has shifted from clarifying the phenomenology to elucidating its underlying mechanisms (Michael and De Bruin, 2015). Many researchers still appeal to mechanisms of inference and/or simulation that are restricted to an individual, but there is also a growing recognition that embodied interaction itself plays a role in such social perception.

### Toward a Relational Hypothesis

We can motivate this interactive approach to the phenomenon of direct social perception from both phenomenological and developmental perspectives, which converge on basically the same relational hypothesis: the conditions underlying social awareness cannot be reduced to an isolated individual; it is rather the other’s intentional and attentional engagement with one’s self that helps to constitute one’s awareness of the other’s presence.

Reddy (2008) has argued for this hypothesis at length from a developmental perspective. She points out that if the other’s mindedness is directly expressed in their embodied interaction with the world, and especially in their engagement with one’s self, then all an infant needs is a predisposition to attend to and pick up the other’s embodied manifestations of mentality. And the infant is surrounded with such manifestations from the moment of birth, in particular the unavoidable manipulations of its own body when it is picked up and carried, fed, cleaned, bathed, and so forth. Accordingly, she contends that infants’ first experience of others is constituted by being the object of their caretakers’ attention (Reddy, 2003, 2005).

The relational hypothesis has also been defended by the phenomenological tradition of philosophy, especially by Merleau-Ponty (1960/1964a,b). He argued that infants’ awareness of other minds develops from within a dyadic state of consciousness, and that our most basic awareness of other minds even as adults is constituted by passive touch. He specifically focused on touch, rather than other perceptual modalities, because the experience of touching one’s own body exemplifies how we can perceive embodiment in both is objective and subjective aspects: while attending to how my right hand is touching my left hand I can experience myself as the active subject of that action, but I can also switch my attention to the left hand as it is being touched and thereby experience myself as the passive object of that action. In this way we have direct experiential insight into how it feels to be the target of someone’s intentional touching (in this case by ourselves).^1^

It is notable that Merleau-Ponty appeals to an element of passivity in his account of intersubjective experience, despite his usual emphasis that perceiving is an active process. Similar to how one needs to adopt a passively receptive attitude to feel one’s left hand as being intentionally touched by the active right hand, a moment of passivity in one’s interaction with another person helps to make the other’s activity appear as intentionally originating from the other. In other words, by temporarily withholding one’s self from being the active center of reference of one’s experience of the interaction one allows the other’s contribution to become present as originating from another autonomous center of reference.

Miyahara (2015) has identified an unresolved issue in Merleau-Ponty’s account of passive touch as the foundation of intersubjectivity. He notes that we can come into tactile contact with many kinds of objects, and merely undergoing a tactile sensation is not sufficient by itself to cause a transition to a conscious experience of another subject’s presence. For example, if an apple happens to fall on my head while I walk under an apple tree, I will passively undergo the tactile sensation of its impact. But that experience is not a case of passive touch: experiencing a mere tactile impact is not the same as a feeling of being touched by someone. Miyahara (2015) suggests that a necessary condition for the latter type of experience is a more continuous form of actual or potential contact. For example, if my arm is continuously stroked by a branch of the tree while it is blown by the wind, I may have the creepy sensation that the tree is touching me.^2^

Nevertheless, we suggest that an illusion of passive touch will normally quickly break down, for example if I occasionally interrupt my passive mode by actively responding to the stimulation. This will reveal that the movements of the branch are not contingent on my actions and are therefore themselves not susceptible of passive touch. Accordingly, we propose that the reciprocity of social interaction, for example during coordinated handshakes, is another essential factor in the constitution of awareness of other minds. Reciprocity also implies that the participants of the interaction share their social awareness, such that one’s awareness of the other is at the same time matched by the other’s awareness of one’s self, i.e., there is a common sense that we are aware of each other.

Given these considerations we propose a relational hypothesis of social perception: *one’s awareness of another mind emerges in a context of co-regulated interaction and is preceded by a passive period of being the autonomous other’s object of attention*. We set out to empirically evaluate this hypothesis in the tactile modality because, as we have discussed, this has been argued to be the primary modality for direct social perception in both developmental and phenomenological terms. Given that the hypothesis is sufficiently general so as to be applicable to infants and adults, we base our analysis on a paradigm involving pairs of adult participants because this enables us to evaluate subjective reports of the quality of their experience of each other.

### The Perceptual Crossing Paradigm

A popular approach to studying the tactile sensorimotor interaction dynamics associated with recognition of the presence of another person was pioneered by Lenay and colleagues (Lenay et al., 2006; Auvray et al., 2009; Auvray and Rohde, 2012). This so-called “perceptual crossing” paradigm was designed to test whether adults are sensitive to the responsiveness of another’s tactile stimulations under the controlled conditions of a minimalistic virtual reality environment, an approach which in turn was inspired by the double TV monitor paradigm that Murray and Trevarthen (1985) designed to demonstrate that infants are sensitive to social contingency during visual interaction with their mothers. The general aim of these paradigms is to allow the controlled study of social interaction dynamics in real time.

In the perceptual crossing paradigm two players in separate rooms are asked to locate each other in a virtual environment, a line that is invisible to them, by using a simple haptic interface. No other form of interaction is possible. They can move their avatars using a computer mouse and they receive a tactile stimulation to their hand whenever their avatar overlaps with a virtual object. There are three objects that can be encountered by a player: (1) the other player’s avatar, (2) the other avatar’s “shadow” – an object that simply copies the other avatar’s movements at a fixed distance, and (3) a static object (see section “Materials and Methods” for more details). All three objects have exactly the same size, which means that overlapping with them gives rise to the same “on” tactile sensation. Nevertheless, the objects afford different forms of interaction: only two out of the three can move, and out of these two only the other’s avatar can respond to being touched because only this situation of perceptual crossing means that both participants receive tactile feedback at the same time. In other words, the shadow object serves as a special kind of distractor because even though it moves identically to the other participant’s avatar, only the latter is potentially responsive. Participants were asked to click a button on the computer mouse to record the moment whenever they judged that they were currently interacting with the other’s avatar. They knew that in addition to another’s avatar they could also encounter two other objects, but they were not informed that one of those objects copied exactly the movements of the other’s avatar. No feedback was provided about click correctness.

The original study by Auvray and Rohde (2012) and several replications found that participants were able to successfully locate each other in the virtual space, with most clicks being on target. However, surprisingly, participants seemed unable to consciously distinguish their partner’s avatar from the moving distractor object: the probability of clicking after making contact with the partner was not significantly different from the probability of clicking after making contact with the other’s shadow. Instead, the correct social judgments could be explained by the relative stability of mutual interaction. Since both participants actively look for each other, they tend to continue interacting when they happen to make mutual contact, while tending to move away from overly stable (likely caused by a static object) and overly unstable (likely caused by a non-responsive object) situations. The solution to the task, i.e., a participant’s sensitivity to social contingency, was thus interactively realized at the collective level of description.

Froese et al. (2014a) implemented a team-based variation of the perceptual crossing paradigm where the pairs of players were asked to cooperate. They found that most teams were successful; on average players were significantly more likely to click in response to a stimulation from the other’s avatar than any other object. In contrast to previous studies this difference remained significant even at the level of individual conditional probabilities. They also found that trials in which both players correctly identified each other led to significantly higher perceptual clarity ratings than when only one player clicked correctly or a player clicked wrongly. Such situations of clear perceptual awareness and joint success tended to be preceded by elevated levels of turn-taking (TT) interaction, which was interpreted as confirming the hypothesis by Froese and Di Paolo (2011) that co-regulation of interaction gives rise to a distinctively social kind of perceptual experience. Clicks in jointly successful trials were most likely to occur within seconds of each other, which is consistent with the possibility that a dyadic awareness of each other was shared between participants.

This dyadic nature of the interaction was further supported by a re-analysis of the sensorimotor time series by Zapata-Fonseca et al. (2016). They revealed that the clustering statistics (Allan factor) of discrete movement events was characterized by fractal scaling, which highlights that the social interaction process operates in a distributed fashion across multiple timescales. Moreover, the complexity matching, defined as the interpersonal similarity between these scaling laws of the clustering statistics, was significantly more pronounced in real pairs of participants as compared to surrogate dyads. This confirms the multi-scale distributed character of real-time social coordination, and extends previous complexity matching results from dyadic verbal conversations (Abney et al., 2014) to embodied interaction dynamics. The finding will not come as a surprise to developmental psychologists who have long recognized the communicative and dialogical potential of even basic embodied interaction (Trevarthen, 1979; Tronick, 1989; Stern, 1998; Reddy, 2008).

A diachronic re-analysis of that original study by Froese et al. (2014b) confirmed the expected relevance of the perceptual crossing paradigm for understanding the development of social perceptual awareness. It seems that the unfamiliar experimental setup forced the adults to implicitly re-learn the embodied skill of social perception, which may provide researchers with an opportunity to study a recapitulation of its original development in adults. In particular, a trial-by-trial qualitative analysis of participants’ free-text subjective descriptions turned out to be roughly consistent with Reddy’s (2003) proposal about the qualitative stages involved in being the other’s object of attention. Initially, participants were more likely to report a self-centered awareness of the other’s presence via being the object of their attention, such as a feeling of being explored by the other, which later on became complemented by descriptions of more dyadic and complex forms of attention, such as exchanging specific patterns of stimulation and spontaneously adopting roles of leader and follower. This trial-by-trial process of implicit learning was accompanied by a general increase in the frequency of clear perceptual awareness scale (PAS) scores and joint clicking success.

The diachronic analysis of phenomenological descriptions by Froese, Iizuka, and Ikegami was consistent with the primacy of passive touch in that descriptions of the other’s attention to the self tended to precede more complex forms of social engagement. However, their qualitative analysis cannot tell us whether the moment preceding recognition of the other was objectively characterized by a relative increase in the passive reception of tactile stimulation caused by the other’s movements. Neither does it allow us to verify if a longer duration of passive touch as such entails a clearer awareness of the other’s presence, or whether coordinated sensorimotor interaction is more important. In this study we addressed these challenges based on a more refined analysis of the sensorimotor data generated by that experiment.

## Materials and Methods

The dataset we re-analyzed was originally reported by Froese et al. (2014a). We therefore only briefly describe the participants and the experimental setup as far as it is necessary to understand the data. Then we introduce the techniques of analysis we applied.

### Participants

Participants were healthy volunteers recruited from acquaintances at the University of Tokyo and at the University of Osaka (*N* = 34). There were 25 Japanese nationals, the rest were from various countries. Six were female. The mean age was 29 years. Teams of participants were created as volunteers became available.

The study protocol was approved by the local ethics research committee of the Graduate School of Information Science and Technology, University of Osaka, and by the local ethics research committee of the Graduate School of Arts and Sciences, University of Tokyo, and has been performed in accordance with the ethical standards laid down in the Declaration of Helsinki. All of the participants gave their written informed consent before taking part in the study.

### Experimental Setup

**FIGURE 1:**
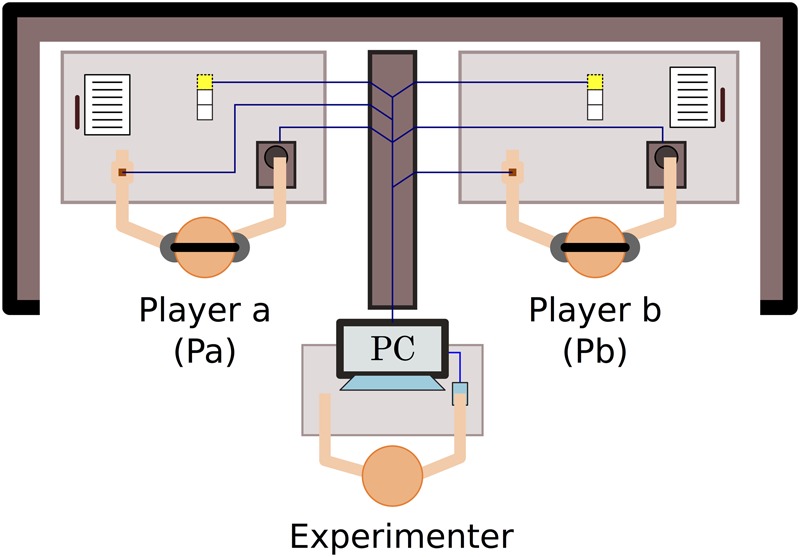
Experimental setup of perceptual crossing paradigm. The two participants can only engage with each other via a human–computer interface that reduces their scope for embodied interaction to a minimum of translational movement and binary tactile sensation. Each player’s interface consists of two parts: a trackball that controls the linear displacement of their virtual avatar, and a hand-held haptic feedback device that vibrates at a constant frequency for as long as a player’s avatar overlaps with another virtual object and remains off otherwise. Three small lights on each desk signal the start, halftime (30 s), and completion of each 60-s trial. Figure originally published in Froese et al. (2014a).

**FIGURE 2:**
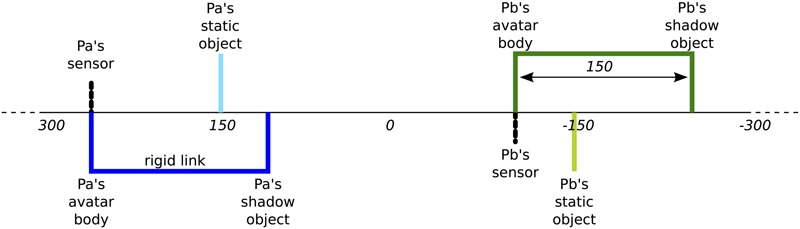
Virtual environment of perceptual crossing paradigm. Players *Pa* and *Pb* are virtually embodied as “avatars” on a line that wraps around after 600 units of space. This virtual space is invisible to the participants. Each avatar consists of a binary contact sensor and a body object. Unbeknownst to the players a “shadow” object is attached to each avatar body at a fixed distance of 150 units. There are also two static objects, one for each player. All objects are four units long and can therefore only be distinguished interactively in terms of their qualitatively different affordances for tactile engagement. No other forms of interaction were possible. Figure originally published in Froese et al. (2014a).

In Froese et al.’s (2014a) version of the perceptual crossing paradigm, two adults are placed in distinct locations such that they cannot perceive each other; their sight is blocked and they wear noise-cancelling headphones (**Figure 1**). Their only manner of making contact is via a simple interface consisting of a trackball that records horizontal movements and a hand-held vibration motor that is either on or off. The trackball is operated with the dominant hand while the motor is held in the other hand. Their movements control the motions of an avatar located in an invisible 1D virtual environment (**Figure 2**). The motor continuously vibrates whenever their avatar overlaps with another object in the virtual space. Position and sensor data were recorded every 10 ms (100 Hz).

The task given to pairs of players was to form a team and to help each other to find each other in the virtual space. They are to click once using the trackball (and only once per trial) in order to signal to the experimenters when they become aware of interacting with the other player; the other player is not aware of the click. No feedback is provided during the experiment. Each pair can interact in a sequence of 15 trials, each with a duration of 60 s. After each trial the experience of the players is evaluated in several ways if they happened to click in that trial. In particular, they were asked to rate the clarity of their experience of the other’s presence at the moment of their click on the basis of a social version of the PAS, based on the PAS that was proposed by Ramsøy and Overgaard (2004). In this scale 1 means having had no experience, 2 means having had an ambiguous experience, 3 means having had an almost clear experience, and 4 means having had a clear experience (**Table 1**).

**Table 1:** Froese et al.’s (2014a) social version of the perceptual awareness scale (PAS) adapted from the PAS by Ramsøy and Overgaard (2004).

PAS	Experience of other’s presence
1	No experience
2	Ambiguous experience
3	Almost clear experience
4	Clear experience

### Analysis of Sensorimotor Trajectories

First, we replicated and extended previous work on measuring the amount of interpersonal coordination that precedes clicks.

The degree of TT was calculated following the method proposed by Froese et al. (2014a). At each time step we classified the state of each player’s behavior in binary terms as either moving (1) or non-moving (0) by evaluating his or her trackball movement (we will refer to these binary movement time series as B1 and B2 for participants Pa and Pb, respectively). Movement was considered to have taken place whenever the change in avatar position d*x* from one time step to the next was bigger than an 8th of the avatar’s length (i.e., 4/8 = 0.5 so that if d*x* > 0.5, 1, else 0). Since avatar positions tend to fluctuate during a player’s “turn” we chose to set a lower limit to the duration of movement pauses so as not to accidentally end up with a turn being divided into micro-turns. Thus, we only set movements to 0 if there was no motion over at least 50 consecutive time steps (500 ms), otherwise they remain set to 1.

In order to determine the differences between players’ activity we applied the logical “Not-And” operator to their movement time series, which resulted in a time series of activity differences *D* (i.e., *D* = B1 Not-And B2). Then, we assigned to each participant their active contribution of this exchange by applying the logical “And” operator and summing the result [i.e., C1 = sum(B1 And *D*); C2 = sum(B2 And *D*)]. The overall TT performance for a given time period was then calculated by multiplying the player’s active contributions. This multiplication means that one-sided situations, in which one player is continuously active while the other is continuously passive, get low TT scores. Finally, we normalized the outcome such that the TT score TT = (4 ^∗^ C1 ^∗^ C2)/*T*^2^, where *T* is the number of time steps. The range of TT is therefore [0, 1], with 0 representing a complete absence of TT interactions and 1 representing a perfect exchange of periods of activity and passivity between the subjects. We analyzed the TT in the 10 s preceding a click (or correspondingly less when the click occurred within the first 10 s of a trial).

The measure of TT interaction that was proposed by Froese et al. (2014a) can tell us whether players were exchanging periods of activity and passivity in an orderly manner, but it does not say much about the similarity of the patterns of activity that were being exchanged. Given that interpersonal synchrony is widely considered to reflect psychological connectedness, we applied measures of movement synchrony, namely cross-correlation (CC) and windowed cross-lagged regression (WCLR). CC is a common measure but can be confounded by auto-correlation, which may lead to inflated measures of interpersonal synchrony, a problem which is avoided by WCLR (Altmann, 2011). High levels of synchrony can mean that both players are moving similarly at the same time or with a lag. We analyzed periods of 10 s with a time lag in the range of [-5, 5] seconds, which means that clicks occurring during the first 15 s of a trial were excluded from CC and WCLR analysis. In total, 28 clicks had to be excluded. We also used WCLR to calculate the windowed time delay yielding the largest CC value, which gives an indication of the most relevant timescales in which synchrony can be measured.

Second, we looked more specifically at the influence of players’ movements on each other’s tactile sensations preceding a click using a measure known as local transfer entropy (TE). This analysis of the whole sensorimotor loop is a significant methodological advance because previous time series analyses of perceptual crossing have only focused on movement by itself, thus leaving the interdependency between movement and sensation underlying meaningful perception (Noë, 2004; Mossio and Taraborelli, 2008) unexamined. TE was originally proposed by Schreiber (2000), and this measure can capture the directional influence from one time series to the other time series. This TE can be also formulated in temporally local form (Lizier et al., 2008), which allows us to calculate TE for specific segments of a time series.

Transfer entropy uses a joint probability distribution from two time series, and to calculate this distribution function empirically from the time series, we need to discretize the time series. The simplest way is to convert the movement time series to a binary sequence is in terms of the directional change of movement, i.e., we label the point in time as a state 1 when it changes, otherwise we turn it into a state 0. The sensory time series was also converted to a binary sequence, i.e., when the haptic feedback turns on or off we label the point in time as a state 1, otherwise it is set to 0 to mark the absence of a change in sensor state. In order to determine the most important timescale of the time series, first we calculated TE by utilizing the whole trial while adjusting the down-sampling rate. We found there was a peak of TE around 50 ms from the movement data to the sensory input data. We therefore took 50 ms as the characteristic timescale and used it for the further analysis of the local TE.

Given that we are interested in determining the sensorimotor signature of social awareness, we related these objective measures with the subjective PAS ratings of the clarity of the other’s presence. In particular, we excluded clicks that were not reported to have been associated with an experience (i.e., PAS 1 or no PAS report), and restricted the data to ambiguous, almost clear, and clear experiences (i.e., PAS 2, 3, and 4, respectively). In addition, we did not further discriminate between the clicks that are associated with these conscious reports in terms of their objective correctness since we were interested in studying the general conditions of the transition to a social experience rather than to a veridical social experience *per se*. The final dataset consisted of 101, 122, and 143 clicks associated with reports of a PAS score of 2, 3, and 4, respectively. Out of these 366 clicks 321 correctly identified the other’s avatar.

### Statistical Analysis

In order to analyze the relationship between the PAS and the movement coordination measures (TT, CC, and WCLR), first we averaged those movement measures with the same PAS, and applied one-way ANOVA followed by *post hoc* Bonferroni test.

In the analysis of local TE, we used Welch’s *t*-test to examine whether the average TE were different before and after a click.

## Results

**FIGURE 3:**
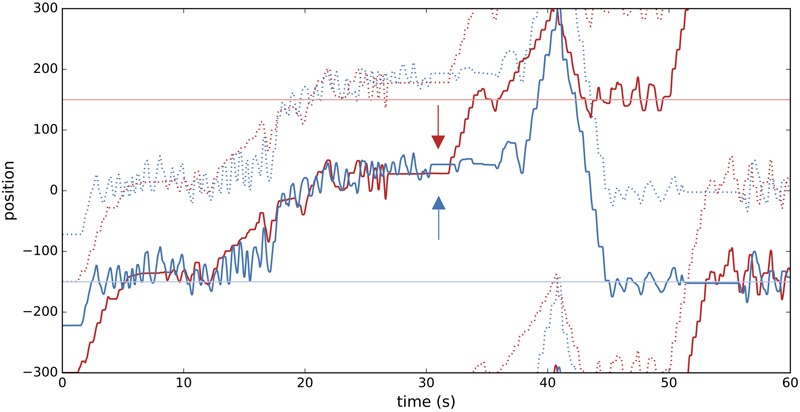
Example of time course of an illustrative trial (E1T1). Thick red and blue lines show the change in position of the avatars of players Pa and Pb, and red and blue dotted lines trace their “shadow” objects. Horizontal lines at *y* = –150, 150 correspond to the position of the player-specific static objects. Red and blue arrows indicate the time of clicking by Pa and Pb, which occurred practically instantaneously (within 0.05 s). After the trial players Pa and Pb reported that their experience of the other’s presence at the moment of the click consisted in a “vague impression” (PAS 2) and a “clear experience” (PAS 4), respectively.

Players’ activity during a typical trial is shown in **Figure 3**. Note the extended period of interpersonal interaction in the first half of the trial, followed by nearly instantaneous clicks by both players. This is followed by disengagement, then a short interaction with their respective static objects, and finally re-engagement just before the end of the trial.

### Qualitative Analysis of Movement Coordination

**FIGURE 4:**
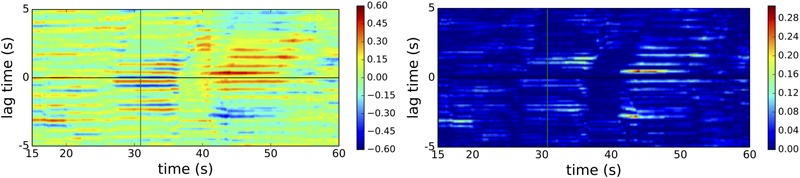
Heat maps of cross correlation (CC, left) and WCLR (right) for an illustrative trial (E1T1). Measures are applied to periods of 10 s. The *x*-axis corresponds to the end point of the time window. The *y*-axis corresponds to the length of the windowed time lag; a positive sign means that behavior of participant Pa can explain that of Pb after the given delay (conversely, a negative sign means that the direction of influence instead goes from Pb to Pa). Thus, it starts at 15 s because a lag time of 5 s means that we compare Pa’s activity from 0 to 10 s with Pb’s activity from 5 to 15 s (or vice versa for a lag time of –5 s). The vertical lines after 30 s represent the nearly instantaneous moment of clicking by both players.

We can use the example trial shown in **Figure 3** to illustrate the CC and WCLR measures (**Figure 4**). It can be seen that CC greatly overestimates the amount of movement synchrony, while WCLR picks out only a few temporal regions. For example, there is a bright blue patch from *x* = 15 to 20 s for time lags of around -3 s. This tells us that during the preceding 10 s periods, starting from 5 to 15 s and ending during the period of 10–20 s of that trial, player Pb leads Pa with a delay of around 3 s. If we check this result against what is happening during that time in **Figure 3**, we can see that indeed player Pb (blue line) leads Pa (red line) beginning around 8 s by inducing the latter to also start oscillating. In the 10 s preceding the clicks, **Figure 3** shows that the direction of influence has become reversed, with Pa staring to oscillate from around 25 s and then pausing, while Pb continues to oscillate until pausing around 30 s. If we compare this with **Figure 4**, we see some bright blue bands following the clicks for time lags of around 2 s, which suggests that in the seconds preceding the clicks Pa’s behavior leads Pb’s behavior.

Nevertheless, it can also be observed that the WCLR method may be confounded when the players happen to move similarly but without interacting directly. For example, it turns out that the highest values in **Figure 4** are given for the period from around *x* = 42 to 50 s for lag times of around 0.5 s, even though **Figure 3** reveals that in the corresponding period starting from 32 s onward the players had already separated and just happened to move in roughly similar ways, with Pa slightly leading Pb but without direction interaction. However, we do not arbitrarily want to exclude such cases of behavioral coordination because they may still tell us something meaningful about the quality of the interaction. After all, one possible reason why these two players continued to move similarly even after spatially disengaging is that they had already become entrained during the first half of the trial.

**FIGURE 5:**
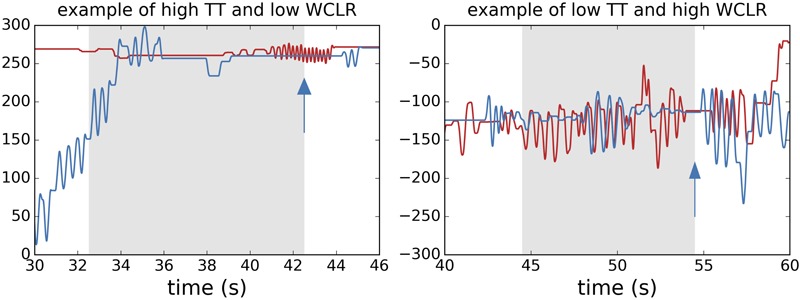
Illustrative comparison between turn–taking (TT) and WCLR measures. For clarity we only plot the change in position of the avatars of players Pa and Pb (blue and red lines). Arrows mark the moment of player Pa’s click. Taking turns by exchanging periods of movement and passivity does not entail a similarity between players’ movement patterns (left, E7T6), and a similarity between players’ movement patterns does not entail a TT interaction (right, E17T15). The values for TT and WCLR for the 10 s before Pa’s click (shaded regions) are 0.49 and 0.015 for the example on the left and 0.0 and 0.18 for the example on the right, respectively. The lag times that yielded these maximum WCLR values were –4.4 (left) and –2.8 s (right).

We note that TT interaction and synchrony can both give high values for a trial when the players exchange periods of activity and passivity whereby that activity is similar in form, too. But they can also be mutually dissociated in other cases. As illustrated in **Figure 5**, players can exchange periods of activity and passivity whereby that activity itself does not have much resemblance (high TT and low WCLR), and players can greatly overlap in their activity but still share a lot of similarity in their movements (low TT and high WCLR). Here we applied the measures to the 10 s preceding a click, and we calculated the WCLR value to be the maximum value from a range of window time lags [-5 s, 5 s].

### Quantitative Analysis of Movement Coordination

Turn–taking interaction and movement synchrony could spontaneously emerge from the interaction dynamics without necessitating any explicit intention to coordinate behaviors or awareness that this is in fact occurring (Froese et al., 2012). In other words, conscious experience of social interaction cannot be reduced to objective measures of coordination; both subjective and objective aspects must be taken into account in an integrated manner.

**FIGURE 6:**
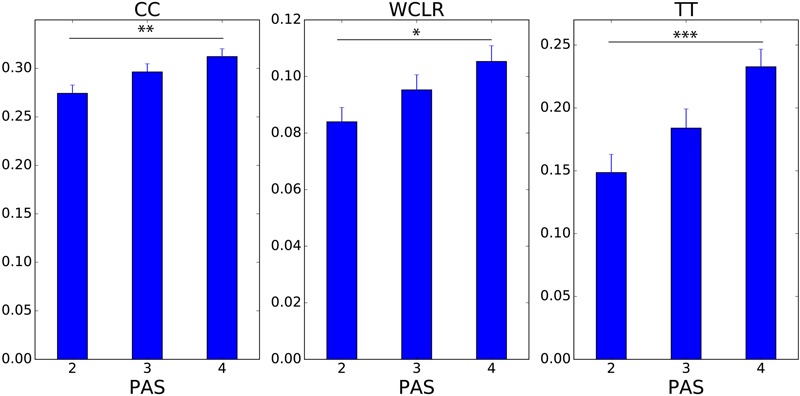
Three measures of interpersonal movement coordination and the clarity of the other’s presence (PAS). We averaged the three measures of interpersonal movement coordination, CC (CC, left), WCLR (middle), and TT (right) scores for each PAS rating. The measures were applied to the 10 s preceding a click. ^∗^*p* < 0.05, ^∗∗^*p* < 0.01, ^∗∗∗^*p* < 0.001 (with Bonferroni correction).

Here, we compared each movement coordination measures with different PAS ratings (CC: 0.27 ± 0.01, 0.30 ± 0.01, 0.31 ± 0.01; WCLR: 0.084 ± 0.005, 0.095 ± 0.005, 0.11 ± 0.01; and TT: 0.15 ± 0.01, 0.18 ± 0.02, 0.23 ± 0.01, with PAS 2, 3, and 4, respectively), and we found that there was difference among different PAS ratings for all the objective measures (ANOVA, *F*(2,337) = 4.969, *p* < 0.01, *F*(2,337) = 3.792, *p* = 0.024, and *F*(2,364) = 8.178, *p* < 0.001 for CC, WCLR, and TT, respectively). Especially we found that all movement coordination measures accompanying with PAS 4 were higher than with PAS 2 (*t*(225) = 3.160, *p* < 0.0.01, *t*(225) = 2.728, *p* = 0.021, *t*(242) = 4.055, *p* < 0.001 for CC, WCLR, and TT, respectively, with Bonferroni correction) (**Figure 6**).

This is an indication that these measures are characterizing some part of the sensorimotor interaction signature of a clear experience of the other’s presence. This seems to suggest that elevated levels of TT and movement synchrony are a common feature of the transition to social awareness during interactions.

### Timescales of Movement Coordination

**FIGURE 7:**
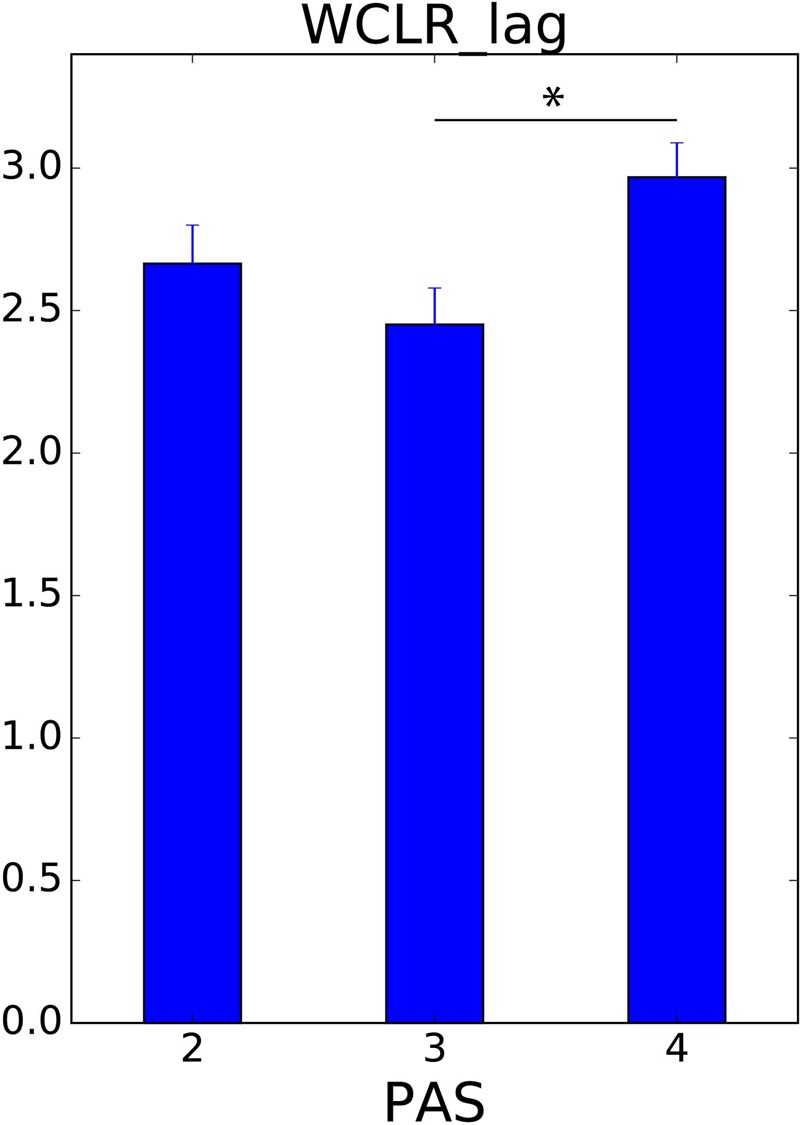
The timescale at which movement imitation is most pronounced (WCLR lag) and the clarity of other’s presence (PAS). We calculated the size of the windowed time lag yielding the maximum value for WCLR and averaged it over the same ratings of clarity of other’s presence (PAS). ^∗^*p* < 0.05 (with Bonferroni correction).

In order to learn more about the timescales in which synchrony of movements is most pronounced, we used WCLR to calculate the delay giving the highest CC value for each trial. Since here we were not interested in which of the two players was leading the interaction, we took the absolute value of the lag times. We related these values with players’ PAS ratings to determine whether some timescales are more relevant for explaining a clearer experience of the other’s presence (**Figure 7**). If Miyahara (2015) is correct in suggesting that a continuous possibility of passive touch is essential for perceiving the presence of the other, clearer awareness should presumably be correlated with a longer timescale of interaction.

Averaged lag time with each PAS rating were evaluated as 2.7 ± 0.1, 2.5 ± 0.1, and 3.0 ± 0.1 s. We found that the average lag time was significantly different among different PAS ratings [*F*(2,337) = 4.395, *p* = 0.013], and especially we found that the lag time with PAS 4 was significantly longer than that with PAS 3 [*t*(240) = 2.915, *p* = 0.012, with Bonferroni correction]. Those findings suggest that phenomenologically more salient forms of movement synchrony are based on a longer timescale of interaction.

### Analysis of Direction of Influence

We used local TE to quantify directions of influence before and after a click at different timescales (a period of 10, 5, and 1 s before a click and 1, 5, and 10 s after a click). The periods of 1 and 10 s were chosen to coincide with the two cognitive scales of Varela’s (1999) three scales of duration of the temporal horizon: (1) basic or elementary neural events (the “1/10” scale); (2) relaxation time for large-scale neural integration of cognitive or perceptual acts (the “1” scale); and (3) descriptive-narrative assessments of the situation (the “10” scale). The 5 s scale was chosen as an intermediate scale that is consistent with the lag times used for the synchrony analyses. It is also of interest as an expression of cognitive events taking place at the “1” scale: spontaneous speech in many languages is organized such that utterances last 2–3 s and short intentional movements (such as self-initiated arm motion) are embedded within windows of this duration (Varela, 1999). We return to this point in the discussion.

**FIGURE 8:**
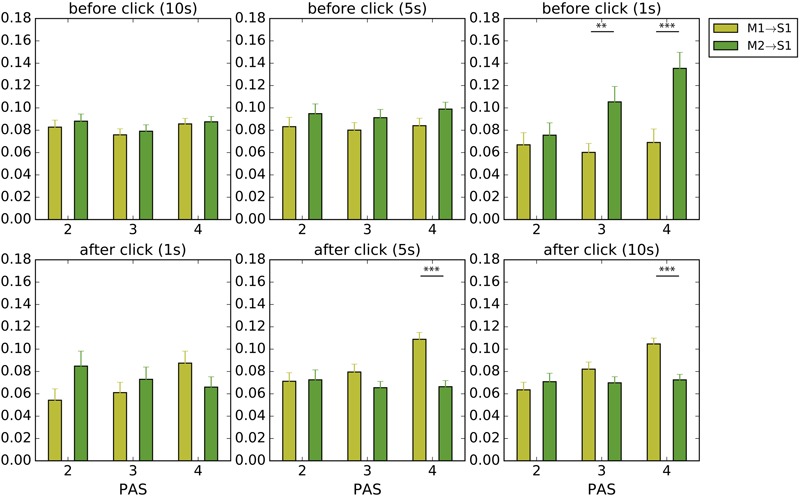
Average local TE to the *self’s* sensations before and after clicks. We analyzed periods of 10, 5, and 1 s before a click and 1, 5, and 10 s after a click. Yellow and green bars indicate how much the movements made by the self (M1), i.e., the player who made the click, and by the other player (M2) contributed to the *self*’s tactile stimulation (S1), respectively. Error bars represent standard error; significance was calculated using the Welch’s *t*-test. ^∗^*p* < 0.05, ^∗∗^*p* < 0.01, ^∗∗∗^*p* < 0.001.

**FIGURE 9:**
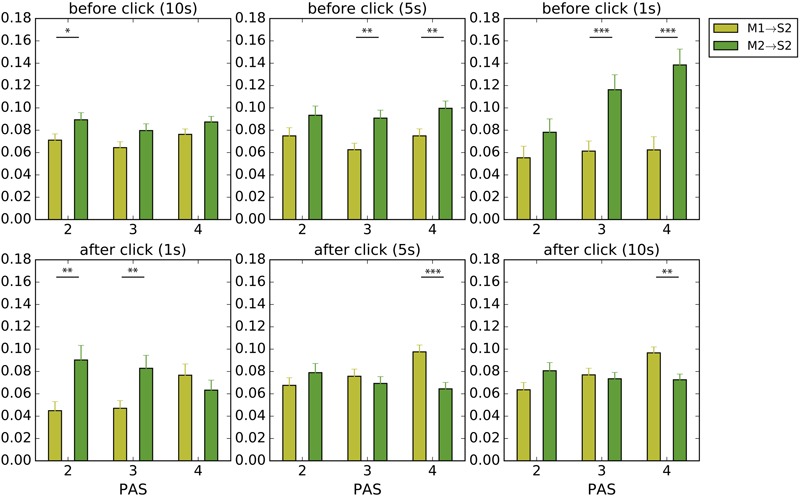
Average local TE to the *other’s* sensations before and after clicks. We analyzed periods of 10, 5, and 1 s before a click and 1, 5, and 10 s after a click. Yellow and green bars indicate how much the movements made by the self (M1), i.e., the player who made the click, and by the other player (M2) contributed to the *other’s* tactile stimulation (S2), respectively. Error bars represent standard error; significance was calculated using the Welch’s *t*-test. ^∗^*p* < 0.05, ^∗∗^*p* < 0.01, ^∗∗∗^*p* < 0.001.

We denote S1 as the “self’s” sensor time series and S2 as the “other’s” sensor time series. Self and other are determined relative to the player who made the click. Who clicks first was not considered here. When one player touches the other, both sensors get activated at the same time (i.e., S1 = S2). They are only different (i.e., S1 ≠ S2) when either player touches the static objects or the shadows. Yet even though this means that **Figures 8, 9** are expected to return similar values for situations of perceptual crossing, we separate M1/2 → S1 (**Figure 8**) and M1/2 → S2 (**Figure 9**) for the sake of clarifying active/passive touch differences.

In support of the hypothesis of passive touch we found that the transition to perception of the other’s presence was characterized by passively received tactile stimulation (**Figure 8**). In general, there tends to be more influence from the other’s movements (M2) on the self’s sensations (S1) compared with the influence of the self’s movements (M1). However, we did not find a continuous period of passive touch, a result that is consistent with our finding that significant lag times are <3 s. A statistically significant heightened influence of M2 on S1 was only observed in the second immediately before the click and only for high PAS ratings [*t*(214.0) = 2.83, *p* < 0.01, *t*(279.9) = 3.54, *p* < 0.001 for PAS 3 and 4, correspondingly).

This pattern is reversed after the click: moments of awareness rated as PAS 3 and 4 are followed by heightened influence of the self’s movements on the self’s sensations (i.e., comparatively more TE from M1 to S1 compared to M2 to S1). Moreover, this difference in influence only becomes significant after a few seconds and remains so until at least 10 s [*t*(274.8) = 5.16, *p* < 10^-6^, *t*(263.2) = 4.47, *p* < 10^-4^, for 5 and 10 s with PAS 4, respectively).

### Analysis of Switch from Passive to Active Touch

This post-click reversal in the flow of influences was unexpected. We considered two plausible explanations. On the one hand, this period of self-generated activity could be an example of reciprocity, in which the self now tries to make its presence clear to the other by providing them with an opportunity for undergoing passive stimulation in return, which would incidentally also stimulate the self’s own sensor due to the situation of perceptual crossing. However, an alternative possibility, which is more in line with the illustrative trial shown **Figure 3**, is that a click marks the end of a bout of close interaction followed by a period of temporary disengagement, in which the movements of the other participant play only a diminished role for the self’s sensations. We therefore redid the analysis shown in **Figure 8**, but this time focusing on the TE from the self to the other’s sensations. The aim is to verify if the self’s movements comparatively increase their influence on the other’s sensations or not (**Figure 9**).

We found that in the periods leading up the self’s click the other’s movements dominated the other’s own sensations (M2 → S2), which is to be expected if the self is mostly passive during the transition to social awareness (M1) and the other is actively moving (M2). After a click the situation becomes more complex. Following moments of clear awareness (PAS 4) the other’s sensations are more influenced by the self’s movements (M1 → S2), and significantly so in the longer post-click periods [*t*(276.8) = 3.93, *p* < 0.001, *t*(263.6) = 3.26, *p* < 0.01, for 5 and 10 s with PAS 4, respectively). This is consistent with the idea that the self returns the feeling of passive touch to the other, which from the self’s perspective involves a transition from passive to active touch, but this possibility is more typical for clear awareness. After less clear experiences (PAS 2 and 3) there tends to be a stronger influence from the other’s movements to other’s own sensations, a trend especially notable for the immediate post-click period (1 and 5 s) and for the least clear experience (PAS 2). This is consistent with the idea that the self disengages from its interaction with the other after making a click, thereby leaving the other alone to generate their own sensations, but this decoupling is more typical for when the other’s presence was not experienced sufficiently clearly.

Both of these situations can be confirmed in **Figure 3**, where the player who first disengages after the clicks (Pa) was also the one who gave a PAS score of only 2, while the other player who apparently would have continued interacting gave a PAS score of 4. These differences in the self’s awareness-dependent post-click behavior, namely the transition from passive to active touch compared with relative disengagement, deserve attention in future studies.

## Discussion

We confirmed Froese and Di Paolo’s (2011) hypothesis that real-time co-regulation of interaction, as measured by TT and movement synchrony, is higher when there is clearer awareness of the other’s presence as measured by PAS ratings. However, although movement synchrony was significant, the relationship between PAS ratings and the movement synchrony was not particularly conspicuous, which suggests that other factors are in play as well. We therefore looked at the role of timescales. We found that the duration of an interaction makes a difference for how it is experienced, because the time lags involved in the most pronounced periods of synchrony were different among different awareness ratings.

We then looked at how participants influenced each other’s sensations via their movements. The results of the TE analysis are consistent with the importance of short timescales. Passive stimulation first becomes notable around 5 s before a click, but only becomes significant in the second before the click. To be fair, this relative reduction in self-generated stimulation may be partially due to the fact that participants often paused their movements in order click. However, even so this pausing does not explain a corresponding increase in influence from the other’s actions to one’s sensations (for instance, the other player could pause as well or disengage altogether). More importantly, it does not explain why the extent to which this passively received stimulation exceeded self-generated stimulation was positively correlated with the reported clarity of the other’s presence. Also, in the original perceptual crossing experiment by Auvray et al. (2009) they reported that the most common cue preceding clicks was “changes in the stimulation without moving” and this is consistent with our result.

We can better make sense of this positive correlation by considering that if one feels a sensation while moving, it is not clear whether this sensation was caused by one’s own or by the other’s movement. However, if one feels a new sensation without moving, then one cannot have caused the sensation. Moreover, the structure of the other’s autonomously generated movements should be more apparent. For example, if I feel a repeated, irregular and yet contingent, i.e., not random, series of sensations it is most likely generated by the other (Lenay et al., 2011). We therefore suggest that participants remained passive during their transition to social perception because this permitted the presence of the other to be most clearly experienced in terms of the other’s autonomous movements being applied to one’s self.

However, in contrast to our expectations, we did not find that a continuous feeling of passive touch is essential, at least not if we interpret continuity to imply periods of more than just a few seconds. The average lag time characteristic of the most pronounced periods of synchrony does not go beyond Varela’s (1999) “1 s” timescale, according to which an integrated cognitive–perceptual act tends to have a maximum duration of 2–3 s. Similarly, passive touch was only found to be statistically significant within one second before a click. Nevertheless, Miyahara’s (2015) speculation that an essential difference between the intersubjective phenomenon of passive touch and other passive tactile encounters resides in the temporal dimension may still be correct. Specifically, he claims: “To experience the other body as *really* involving the other agent, we must experience it as an actual or potential constituent of passive experience in an ongoing, continuous manner” (p. 31).

And indeed the final moment of clear passive touch was embedded within a longer coordination, in which either player could always *potentially* be the receiver of passive touch and which may already have included several *actual* instances of passive touch in the form of TT. Presumably this preceding period of co-regulation ensures the success of interactively coordinated symmetry breaking into the complementary roles required for passive touch. Agent-based models confirm that TT and movement synchrony can be analyzed as spontaneous cooperative and co-creative processes, and they indicate that changes in the extent of mutual predictability might play a role in the coordinated timing of role switching (Ikegami and Iizuka, 2007). Future studies could try to analyze changes in relative predictability using human data.

One limitation of the current study is that it was only applied to adult interactions. Nevertheless, we suggest that our methods could be applied to sensorimotor data from developmental studies with the aim of analyzing preverbal infants’ initial transitions to social awareness. The results are at least consistent with the theory that infants’ first form of primitive social awareness is based on their being the object of other’s attention. They also support the claim that passive touch continues to be operational in adulthood within a larger repertoire of social skills. For example, we cannot tickle ourselves but depend on others to tickle us, so there may still be something special about passive touch even in adulthood. Nevertheless, the relative importance of passive touch for adult social perception is debatable. Even if infants’ social perception is primarily constituted in this relational manner in the tactile modality, as adults we can perceive others as subjects in their own right without being touched by them and without even being the object of their attention. How passive touch could develop into this more generalized capacity is an interesting open problem.

We speculate that once infants develop their visual awareness they tend to experience themselves as being the object of the other’s attention in both the tactile and the visual modality, and that this repeated multimodal association enables them to perceive the other’s presence even when being their object of attention in the visual modality alone. The same may apply to other modalities, such as audition. Presumably, the subsequent development of the capacity for joint attention, which makes infants aware that the other’s attention can also be directed at other objects than the self eventually enables them to perceive the presence of others by only perceiving their engagement with the rest of the world in general. A recent extension of the perceptual crossing paradigm designed to investigate the interaction dynamics underlying mutual awareness of a shared object (Deschamps et al., 2016) could perhaps be adapted to evaluate these ideas in an experimental manner.

## Conclusion

Developmental and phenomenological approaches to embodied cognition have converged on the relational hypothesis that being the other’s object of attention, as exemplified by the phenomenon of passive touch, is the most basic form of awareness of other minds. We evaluated this hypothesis quantitatively by re-analyzing the sensorimotor time series of a perceptual crossing experiment, in which pairs of players were tasked to cooperatively coordinate their movements so as to locate each other in a minimal virtual environment using haptic interfaces, and to identify and subjectively evaluate the moment they became aware of the other’s presence.

We found that the transition to clear awareness of the other’s presence coincides with pronounced moments of passive touch. To our knowledge this is the first time that the relational hypothesis has been supported with quantitative results. In addition, our time series analysis enabled us to extend this hypothesis into new directions. We proposed that elevated levels of sensorimotor coordination enhance one’s awareness of being the other’s object of attention. On our view, a salient moment of passive touch depends on spontaneous symmetry breaking of interpersonal movement coordination and therefore emerges out of a more extended co-regulation of behaviors. This makes reciprocity another essential factor in our account. We suggest that developmental and phenomenological versions of the relational hypothesis have underestimated the role of mutuality, as exemplified by the surprising finding that the clearest social experience tends to be associated with players switching roles in a transition from passive to active touch such that one individual’s awareness of the other transforms into a dyadic awareness of each other.

## Author Contributions

HK did the main analysis of the experiment using TE and CCs. TF helped to write the manuscript, especially the philosophical background of the paper. MO initiated and contributed to the analysis of the experiment and the discussion of the results. HI contributed to the analysis of the paper, and developed the TT measures. TI helped in writing the manuscript, especially the main findings of the paper, and contributed to the analysis.

## Conflict of Interest Statement

The authors declare that the research was conducted in the absence of any commercial or financial relationships that could be construed as a potential conflict of interest.

## Footnotes

1 Other perceptual modalities do not offer this possibility, e.g., of directly seeing myself looking at my own body, which is why they are argued to be secondary. However, it must be acknowledged that we can nevertheless perceive the presence of other subjects visually and even do so while they are not attending to us. We return to the problem of generalizing the capacity of social perception in the section “discussion.” For the moment we simply note that it is still the case that when one is aware of being looked at one tends to experience a particularly strong presence of another subject.

2 This example shows that the direct social perception thesis is not committed to the infallibility of experience. Even if I have the experience that someone is touching me, this may actually turn out not to be the case. On the other hand, it is also not the case that fallibility entails that perceptual experience never directly involves its object of perception. The details of this philosophical issue go beyond the scope of this paper (see Beaton, 2013).
